# An uncommon mimicker of renal malignancy: IgG4-related disease

**DOI:** 10.1186/s12894-023-01304-8

**Published:** 2023-08-12

**Authors:** Ivan Thia

**Affiliations:** FSH: Fiona Stanley Hospital, Murdoch, Australia

**Keywords:** Case report, IgG4-related disease, Renal pseudotumour

## Abstract

**Background:**

Increased usage of cross sectional imaging for a variety of indications, in particular CT imaging, has led to an increased detection of renal and ureteric masses. Benign ureteric masses are rare, with 95% of identified tumours consisting of transitional cell carcinoma (TCC). IgG4-related disease is a recognised clinical systemic autoimmune, inflammatory condition with a propensity for multi-organ manifestation. Nephritis and pseudo-tumour formation can occur when kidneys are involved. Ureteric involvement is more rare.

**Case presentation:**

Forty nine-year-old Korean male was found to have an incidental invasive renal pelvis mass during investigation for chronic back pain and fatigue. Appearance of the tumour was consistent with an invasive malignancy, and consensus from multidisciplinary meeting was to have the tumour removed. Procedure involved a prolonged open surgery with reconstruction of contralateral renal blood supply and was complicated by a long recovery process. Final histopathology confirmed IgG4 renal pseudo tumour diagnosis.

**Conclusion:**

IgG4-related disease is a rare but potentially morbid disease that can mimic various cancers, including lung, pancreas and renal malignancies. A high index of suspicion is required to accurately diagnose this condition, through a targeted history taking, examination and investigation which should include biopsies. Failing to do so may result in unnecessary procedures being performed and exposing a patient to its associated risks.

## Background

Increased usage of cross sectional imaging for a variety of indications, in particular CT imaging, has led to an increased detection of renal and ureteric masses. Benign ureteric masses are rare, with 95% of identified tumours consisting of transitional cell carcinoma (TCC) [1]. IgG4-related disease is a recognised clinical systemic autoimmune, inflammatory condition with a propensity for multi-organ manifestation. Nephritis and pseudo-tumour formation can occur when kidneys are involved. Ureteric involvement is more rare.

This case report details a rare but increasingly important differential for renal and ureteric tumours as its early diagnosis can help patients avoid a needless procedure and be managed well with steroid therapy.

## Case presentation

**Fig. 1:**
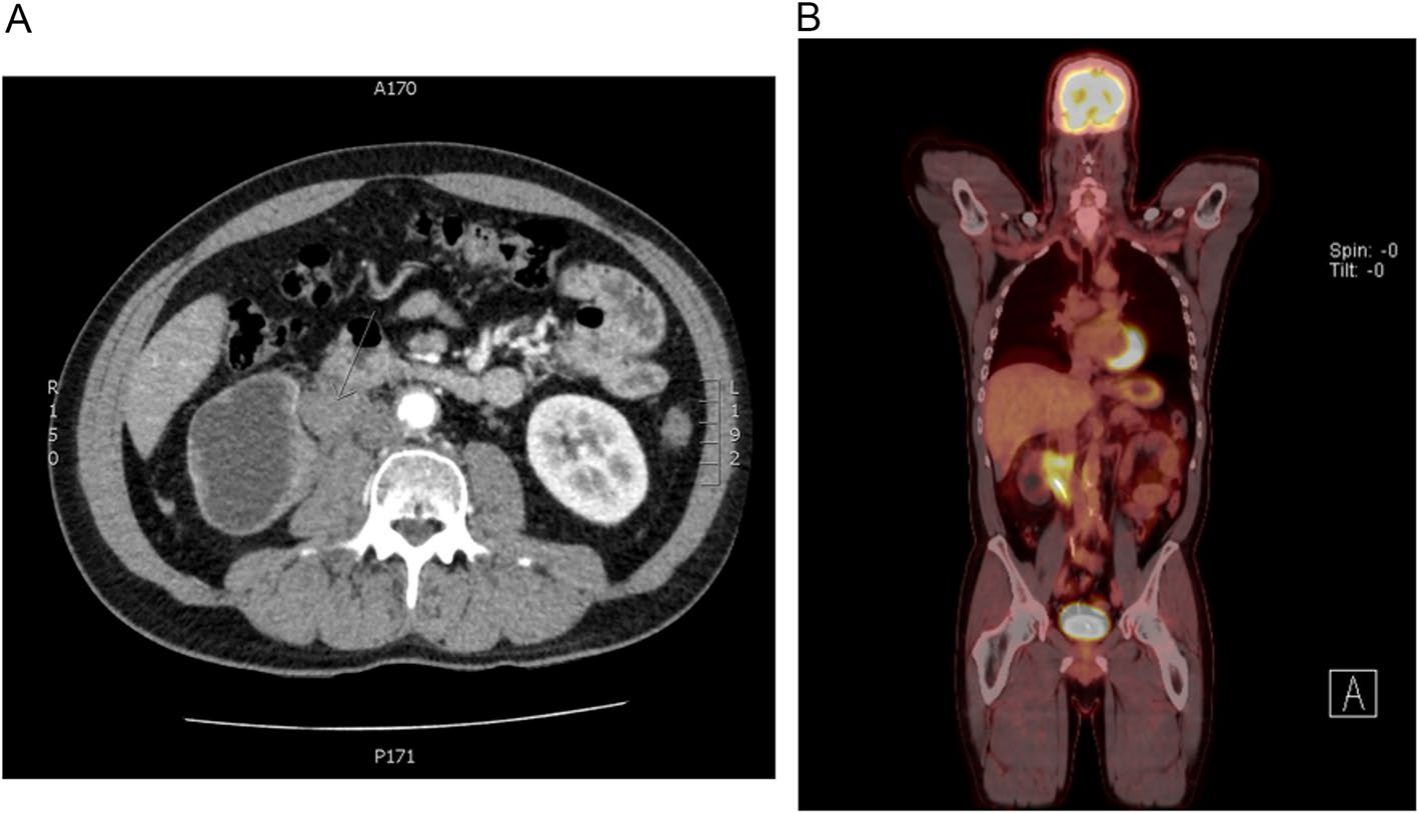
**A** CT imaging demonstrating the renal pelvic mass lesion, associated poor drainage and atrophy of the ipsilateral kidney. **B** PET imaging demonstrating intense FDG avidity of lesion in question

A 49-year-old gentleman from Korea was referred by his GP for further assessment and management when a CT scan performed for investigation of chronic back pain and fatigue demonstrated an incidental finding of a right renal pelvis mass causing compression of the proximal ureter and resultant hydronephrosis and renal atrophy (Fig. 1A). He was otherwise well, with past medical history including obstructive sleep apnoea, hypertension and being a current smoker. Further investigation with a ureteroscopy did not yield results as a dense proximal ureteric stricture precluded proper examination of the renal pelvis, with urine cytology collected being atypical and inconclusive.

With a PET scan demonstrating that the lesion was FDG-avid, and concerns regarding invasion of the tumour into the inferior vena cava and encasing the contralateral renal vein, a difficult decision was made to proceed with an open right radical nephroureterectomy, caval exploration and tumour removal, as well as resection of the left renal vessels with graft reconstruction (Fig. 1B). The local vascular team was invited into the operation as a combined surgical intervention.

The surgery was difficult, with tumour densely adherent to other retroperitoneal structures, and multiple abnormal lymph nodes removed along with the specimen. Recovery of the patient was slow, complicated by ileus, wound infection and clot urinary retention requiring blood transfusion and bladder washouts. During the initial phase, the patient was also suffering from oliguria and a severe renal failure that thankfully did not require intermittent dialysis. Serial ultrasound doppler of the contralateral kidney demonstrated patent grafted renal vessels with no impediment to renal blood flow. All in all, the patient spent a month in hospital and was discharged home when well. His subsequent recovery was uneventful.

**Fig. 2:**
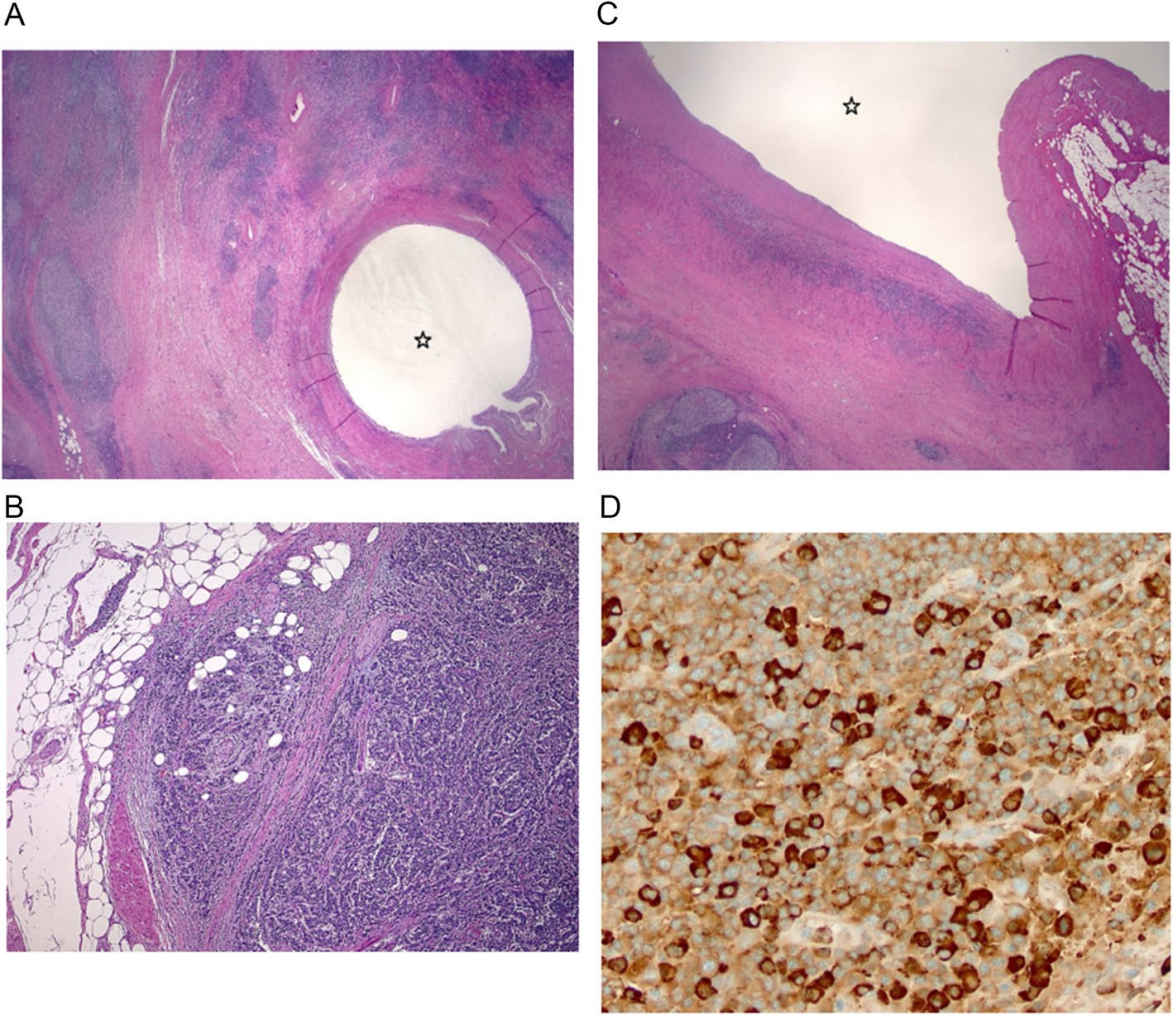
**A** Histopath slide showing ureter surrounded by inflammatory cell infiltrate and fibrosis. **B** Inflammatory infiltrate includes lymphocytes and plasma cells, involving small veins and venules. **C** Infiltrate also extends into inferior vena cava. **D** Immunohistochemical staining for IgG4 highlights abundant plasma cells, in keeping with IgG4-related disease

Histopathology from multiple sections of ureter and renal parenchyma show an expansile fibro-inflammatory process composed of dense lymphoplasmacytic infiltrate along with many lymphoid follicles with prominent germinal centres. Many areas show predominant population of mature plasma cells (MUM1 and IgG4 immunostaining), with the stroma being fibrotic and demonstrating a storiform pattern. Periureteric and renal hilar areas show conspicuous obliterative phlebitis. Immunostaining for smooth muscle and ALK-1 are negative. (Fig. 2A-D). Local IgG4:IgG ratio is 75, confirming an IgG4-related disease process.

**Fig. 3:**
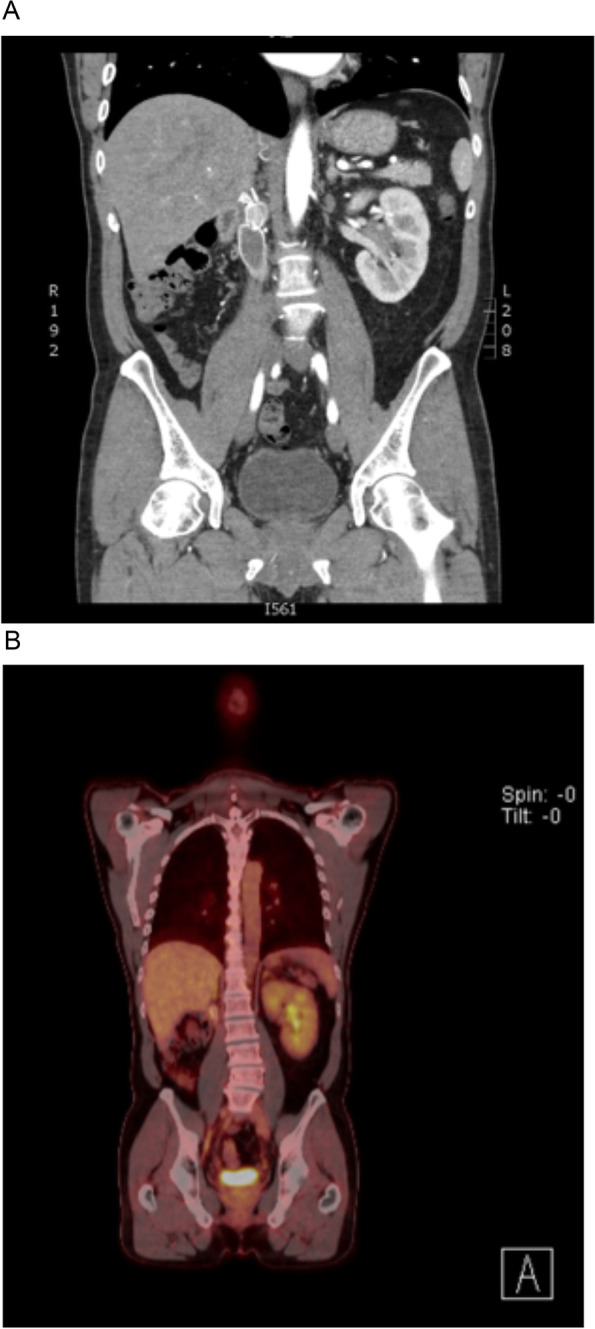
**A** Post-operative CT. **B** Post-operative surveillance PET scan. Both demonstrating complete resection of tumour with no residual disease activity

Follow up with Nephrology and Vascular teams proved uneventful. This gentleman has ongoing follow up with Immunology for imaging PET surveillance looking for potential recurrence of disease. This has so far been negative (Fig. 3A-B). Given the complete resection of the lesion in question and no further deposits detected on scans, as well as serum IgG4 levels being normal post-operatively (1.26 g/L), the decision was made to pursue active surveillance rather than treatment with corticosteroids.

## Discussion and conclusion

IgG4-related disease is a heterogenous group of manifestations that are immune-mediated and fibro-inflammatory in nature. It typical involves multiple organs, with four classic phenotypes of pancreatico-hepato-biliary disease, retroperitoneal fibrosis with or without aortitis, head and neck disease, or classic Mikulicz disease with or without systemic involvement [2].

Incidence of disease is 0.28–1.08/100,000 population, with a slight predominance in middle to older aged man [2]. However, head and neck manifestations are more common in the younger and female population. The asian population also has a slightly higher baseline IgG4 levels of unknown significance.

On histology, affected tissue often display dense infiltrates of predominantly IgG4 positive plasma cells. These are arranged in a storiform, fibrotic pattern and is associated with fibrosis, obliterative phlebitis and eosinophilia. The natural disease progression tend to result in multiorgan involvement (60–90% of diagnosed cases), causing lymphadenopathy and subacute development of various mass lesions in the affected organs, as was the case in the patient discussed [3].

Treatment of this condition is difficult as well, with no optimal treatment guidelines established. All patients with active, symptomatic IgG4-related disease should undergo treatment with glucocorticoid and immunosuppressive therapy such as with the use of rituximab, azathioprine or mycophenolate [4]. Relapsing disease should be re-challenged with glucocorticoid based therapy, with an aim for subsequent glucocorticoid-sparing immunosuppresive maintenance therapy. Other treatment options such as abatacept has been shown to be efficacious in managing treatment resistant disease [4].

IgG4-related disease is a rare but potentially morbid disease that can mimic various cancers, including lung, pancreas and genitourinary malignancies. A high index of suspicion is required to accurately diagnose this condition, through a targeted history taking, examination and investigation which should include biopsies. Failing to do so may result in unnecessary procedures being performed and exposing a patient to its associated risks.

## Data Availability

Single patient data and material either included in current manuscript or available for easy retrieval if required and can be provided from RPH hospital database post publication.
